# Physiotherapy Methods Applied in the Prevention of Functional Loss Associated with Human T-Lymphotropic Virus 1 Infection: An Overview

**DOI:** 10.3390/idr15050048

**Published:** 2023-08-31

**Authors:** Izabela Mendonça de Assis, Bianca Callegari, Maisa Silva de Sousa

**Affiliations:** 1 Center for Tropical Medicine, Federal University of Pará, Belém 66055-240, Brazil; maisasousa@ufpa.br; 2 Institute of Health Sciences, Federal University of Pará, Belém 66055-240, Brazil; callegari@ufpa.br

**Keywords:** human T-lymphotropic virus 1, problems and exercises, physical therapy modalities, review

## Abstract

To achieve the objective of this study, we conducted a narrative review on physical therapeutic modalities applied to prevent functional losses associated with human T-lymphotropic virus 1 (HTLV-1) infections to promote health education and viable and accessible alternatives in the development of health education technology adapted to the home environment. This study comprised a qualitative stage of theoretical development to construct a digital booklet with an observational basis based on studies that reiterate themes about educational technologies as tools to conduct a home protocol of guided exercises without the direct supervision of professional physical therapists. Results indicate a lack of research on the development of health education technologies to assist patients with HTLV-1 without tropical spastic paraparesis or HTLV-1-associated myelopathy/tropical spastic paraparesis (HAM/TSP). We believe that this narrative review can initiate a theoretical framework to conduct a home exercise program aimed at people with HTLV-1 who have subtle symptoms, and also at people without the clinical definition of HAM/TSP, helping to train human resources for care and research on the subject and increase scientific production in physical therapy.

## 1. Introduction

The human T-lymphotropic virus 1 (HTLV-1) is a retrovirus in the Retroviridae family that affects human blood T lymphocytes and can cause neurological disorders. This infection is characterized by silent, long-term persistence in the host. Despite its irregular distribution, estimates suggest that at least 20 million people are infected with HTLV-1 worldwide [1,2,3,4,5], whereas, in Brazil, about two million people live with the infection, but its distribution is heterogeneous and varies geographically [3,6].

The development of serious diseases has been pointed out in association with the virus, such as adult T-cell leukemia/lymphoma (ATL) and tropical spastic paraparesis or HTLV-1-associated myelopathy/tropical spastic paraparesis (HAM/TSP). The latter is characterized by the installation of classic motor disabilities in patients and the slow, progressive, and non-remitting inflammation of the spinal cord, which affects 4 to 5% of infected subjects, causing more proximal motor weakness, spasticity of the lower limbs (LLLL), pain and bladder, intestinal and sexual dysfunctions, and, consequently, functional limitations such as impaired walking, ascending and descending stairs, washing, dressing, and urinary continence [7].

Faced with these significant motor disabilities, physical therapy has been prescribed for neurological complications associated with HTLV-1 because it improves functional status, reduces symptoms, and positively impacts patients’ quality of life [8,9,10]. Considering the importance of implementing physical exercise programs, the development of protocols that can be performed at home provides an alternative for treatment and continued care to resolve health conditions. Professionals must constitute methodologies that successively stimulate patients, as well as the teaching–learning process. Strategies to encourage adherence and motivation are fundamental for the success of treatment performed at home without the direct guidance of a health professional [11].

Such protocols are envisaged as auxiliary strategies for patients with difficulties attending rehabilitation centers as they propose to enable their performance with the use of low-cost materials, promote autonomy and confidence, resume social roles, and seek to provide general data of health conditions associated with HTLV-1 in this population since the levels of evidence and the strength of recommendation for these protocols are yet to be well-established.

Observing the presence of neurological signs and symptoms in HTLV-1 carriers living in the municipality of Belém (PA) [12] in unfavorable socioeconomic conditions, alongside the greater involvement of older women in relation to their presence, their prevalence among the Brown/Black population [13,14,15,16,17,18], as well as the limitations of access to vacancies in therapeutic programs regulated by the Unified Health System [19], the use of new interventions (such as the home exercise program) to strengthen patients’ muscles, improve their flexibility and joint mobility, adjust their postural disorders, and enable economic and cognitive access to other platforms would positively impact these individuals’ quality of life.

Therefore, it is important to conduct a narrative review on how and which physical therapy modalities are applied to prevent functional losses associated with HTLV-1 that can contribute to a safe and supported home clinical practice and highlight new paths in the production of health education technologies.

## 2. Methods

This is a qualitative narrative literature review with an observational basis. This research was submitted to the Ethics Committee for Research Involving Human Beings (CEP) of the Oncology Research Center of the Federal University of Pará, following the Declaration of Helsinki and the norms of Resolution 466/12 of the National Health Council regarding research on human beings (CAAE: 36688020.1.0000.5634).

To conduct this review, the guiding question was first identified, descriptors were selected, and inclusion and exclusion criteria were constituted. Then, the following steps were taken: the sample was selected by searching databases, the information extracted from the selected works was summarized, studies were evaluated, results were interpreted and discussed, and, finally, the review was described and the produced knowledge was synthesized [20].

The guiding question of this study was “What physical therapy approaches can help people affected by HTLV-1 with problems of rehabilitative interest and how?” The construction of the question involved the acronym PICO [21], i.e.,: P for population (people affected by HTLV-1); I for intervention (physical therapy approaches of preventive and rehabilitative interest); C for control (comparison terms were ignored); O for results (functional, quality of life, and perceived pain improvement and hypertonicity reduction).

The following databases were used: Scientific Electronic Library Online (SciELO); Latin American and Caribbean Literature in Health Sciences (Lilacs); US National Library of Medicine (PubMed); and Google Scholar. Data were collected in institutional repositories of theses and dissertation. The inclusion criteria were: (a) articles with full text; (b) drafts in Portuguese or English; (c) exercise protocols aimed at HTLV-1 carriers. No restrictions were set on the sample to maximize search results. The following were excluded: (a) monographs; (b) annals of events; and (c) duplicates. Data were extracted by a standardized form containing information on the method, sample, intervention, markers, and outcomes of the chosen studies.

The following controlled descriptors (indexed to the MeSH terms) were used: human T-lymphotropic virus 1; problems and exercises; physical therapy modalities; Boolean operator “E”. These descriptors in English were also used: “Problems and Exercises”; “HTLV-1”; “HTLV I Associated Myelopathy”; “Physiotherapy Modalities”. A combination of descriptors with the Boolean operator “AND” was used for each of the selected databases due to their specific characteristics, with the guiding question and established inclusion criteria as the guiding principle of this search, without the use of filters.

**Figure 1:**
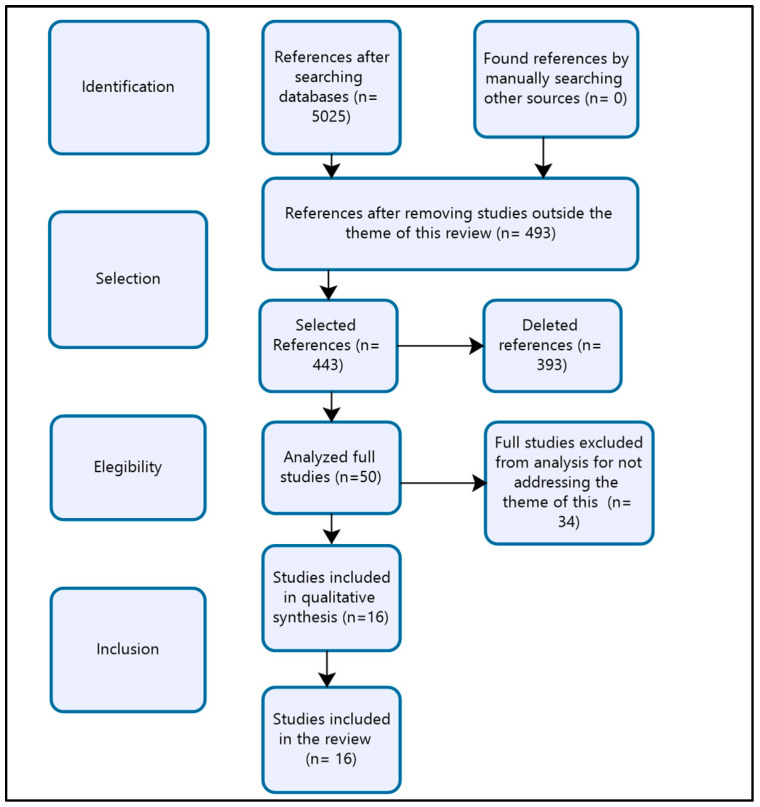
Flowchart of the process of identification, selection, and inclusion of studies.

The search was carried out using online access and considering the period from 2006 to 2022. To select the studies, the recommendations of PRISMA [21] were followed (as shown in Figure 1). In total, 16 studies were included in this review.

## 3. Results

Of the sixteen chosen studies, thirteen are scientific articles, two are doctoral theses, and one is a master’s dissertation. The types of studies in this review include six analytical interventions, two literature reviews, a case report, a short communication, two theses, and a dissertation with an observational descriptive study.

Following the chronological order of the selected study (Table 1), we observed that their results indicate several modalities of physical therapy and specific exercises as promising to treat symptoms associated with HTLV-1. Klautau et al. (2020) [22] showed that the Pilates modality reduced patients’ pain, improved quality of life and trunk balance in wheelchair users, and shortened the progression of lower limb spasticity.

In urinary incontinence associated with the virus, results also point to reduced symptoms after the implementation of behavioral therapy, kinesiotherapy, and electrical stimulation, increasing perineal strength and improving urodynamic parameters (Andrade et al., 2016) [23].

Regarding the evaluation, the use of ICF codes showed a greater functional independence and quality of life in patients after therapy, emphasizing the effectiveness of physical therapy in controlling spasticity and the value of ICF as a tool to analyze spasticity in patients with HAM/TSP (Rodrigues et al., 2015) [24].

PNF increased lower limb ROM, reduced hypertonia/spasticity, and restricted ambulation (Costa et al., 2018) [9]. Regarding adjuvant treatments, repetitive transcranial magnetic stimulation may decrease spasticity and pain in patients with HAM/TSP, but failed to influence patients’ muscle power and quality of life. This intervention can be used as adjunctive therapy in patients with a clinical diagnosis of HAM/TSP (Amiri et al., 2014) [25].

In comparative studies between PNF and outpatient physical therapy, results point to a decrease in low back pain and an increase in functional independence in both treatments (Britto et al., 2014) [26].

The findings of the literature reviews address the importance of implementing specific exercise programs that seek to improve flexibility, range of motion, muscle strengthening, and postural control, as well as the discussion about perspectives to develop knowledge in the area (SÁ et al., 2015; Lannes et al., 2006) [8,27].

Figueiredo et al. (2013) [28] evaluated the impact of a muscle-strengthening program in therapeutic activities on the functional performance of patients with HAM/TSP and found that the program, lasting eight weeks and performed thrice a week for 50 min per session, improves functional performance measures.

By assessing balance in people with HAM/TSP, Patrício et al. (2020) [29] point out that this population has a high balance deficit, and that the use of tools available at no additional cost in outpatient clinics, such as free software for movement analysis (e.g., CYMOB), help identify movement pattern discrepancies and analyze gait performances. Arnaut (2014) [30] also contributed to the investigation on the use of Nintendo Wii as virtual therapy in the treatment of pain and quality of life, positively impacting these two variables.

Mota (2017) [19], who evaluated the impact of a home exercise program with and without supervision on functional mobility and pain in people with HAM/TSP, observed that the home exercise program benefited participants’ mobility and functionality in both supervised and unsupervised groups.

Facchinetti (2013) [31] evaluated the effects of a home exercise program and its adherence rate in individuals with PET/MAH and found that the proposed program effectively improved some disabilities and the quality of life of individuals with PET/MAH.

For Vasconcelos et al. (2019) [32], the subtle balance impairment in patients without defined HAM/TSP (i.e., undetected in clinical scales) suggests that these patients may be between healthy patients and HAM/TSP and, thus, may show a risk of developing severe imbalance postural control.

Finally, for Costa et al. (2022) [34], balance severity directly relates to the degree of signs and symptoms of HAM/TSP, and Almeida et al. (2022) [33] claim that the clinical evaluation of this population should include postural stability interventions during rehabilitation programs.

## 4. Discussion

Although Brazil has a prominent place in scientific production in physical therapy for people with HTLV-1, this production is still in its infancy, mainly testing therapeutic procedures.

A scientometric study on the subject only found 68 studies involving physical therapists, 21 of which were interventional [35]. The most tested therapeutic resources were individual and group functional exercises, including Pilates, home exercise booklets, virtual therapy, and proprioceptive neuromuscular facilitation.

Therapeutic exercise protocols must be improved, especially regarding dosage and progression. On the one hand, individualized exercises, as in PNF, positively reduce spasticity and improve movement control and functionality levels, but they are isolated cases, as in Costa et al. (2018)’s work [9], which reported five cases of patients with HAM/TSP which, thus, may have a higher cost. Group exercises are more accessible, may include a professional assisting a group (which reduces costs), and include the aspect of sharing experiences among peers, as in Klautau (2020)’s work [22], which carried out a pilot study with eight patients divided into two groups, one of wheelchair users and the other with gait impairment. As in Mota (2017) [19] and Facchinetti (2013) [31], home exercises stimulate autonomy for self-care and generate access opportunities for those who are unable to participate in outpatient services.

In view of this, the three modalities of therapeutic exercises should be tested in randomized clinical trials with a larger sample size and more detailed protocols, considering a reasonable follow-up time to enable the measurement of a larger effect size, produce better levels of recommendation, and provide greater security in its reproducibility.

Home care has become one of the main pillars of providing services at different levels of health as it meets the needs of patients with chronic health conditions, improves quality of life by controlling signs and symptoms, and decreases the risk of complications. Thus, the effectiveness of the practice of home exercises after an injury demands that patients, family members, and caregivers understand the importance of therapy, thus reaching an adequate process with greater possibilities of good results [36].

Studies have been using the home approach for physical exercise, considering feasibility and long-term maintenance [37,38,39,40,41], with a more accessible approach to exercise plans that can be performed at home and without the use of special equipment. Home exercise programs are widely used as an alternative strategy for patients with different conditions, such as Parkinson’s disease [42], traumatic spinal cord injury [43,44,45], multiple sclerosis [46,47], Huntington’s disease [48], strokes [49,50], post-polio syndrome [51], cardiovascular diseases [52], etc.

Home exercises remove the need for accessibility to training facilities [53], is cost-effective [54,55,56], and reduces barriers to commuting time [54]. Moreover, standardized home exercise protocols guided by socio-educational materials such as booklets have been found as effective treatments of chronic degenerative diseases, stimulating individuals’ autonomy to manage their condition [57,58,59,60,61].

Thus, gathering evidence with a broad review should precede actions that help prevent functional declines associated with HTLV-1, such as the implementation of public health programs aimed at HTLV-1 carriers without defined HAM/TSP.

## 5. Conclusions

More studies on physical therapy modalities aimed at people affected by HTLV-1 must be developed and tested in randomized clinical trials with a larger sample size and more detailed protocols. We believe that this narrative review can contribute to a safe and evidence-based home clinical practice and point out new paths in the production of health education technologies that are sensitive to the reality of this population.

## Figures and Tables

**Table 1:** Synthesis of selected studies in the narrative review.

#	Title	Authors and Year	Intervention	Results	Conclusions
1	Pilates exercise improves the clinical and immunological profiles of patients with human T-cell lymphotropic virus-associated myelopathy 1: A pilot study	Klatau et al., 2020 [22]	This pilot intervention study sought to evaluate the effects of serial Pilates exercises on the clinical and immunological profile of patients with HAM/TSP. It evaluated eight patients aged between 39 and 70 years (two men and six women), two wheelchair users, and six individuals with impaired gait. Patients underwent 20 Pilates sessions over 10 weeks. Data were collected in three moments (beginning of the study, after the Pilates sessions, and after 10 weeks without Pilates) to assess pain, spasticity, motor strength, balance, mobility, functional capacity, and quality of life and quantify levels of the cytokines IFN-γ, IL-10, and IL-9.	After the Pilates sessions, pain, static and dynamic balance, trunk control, mobility, and quality of life significantly improved, simultaneously and significantly reducing the serum levels of the cytokines IFN-γ and IL-10. However, after 10 weeks without Pilates, pain and mobility regression significantly increased, with no changes in strength, spasticity, or functional capacity in any of the studied periods.	Results suggest that Pilates can be a promising auxiliary physical therapy for patients with HAM/TSP.
2	Effects of physical therapy in the treatment of neurogenic bladder in patients infected with the human T lymphotropic virus 1 (HTLV-1)	Andrade et al., 2016 [23]	An open, uncontrolled clinical trial aiming to evaluate the effectiveness of physical therapy for urinary manifestations in patients with lower urinary tract dysfunction associated with HTLV-1. In total, 21 patients were evaluated at the physical therapy clinic at the University Hospital, Bahia, Brazil. The intervention consisted of combined therapy, including behavioral therapy (with health guidelines), kinesiotherapy (with specific exercises for the pelvic floor to improve the contractility and resistance of muscle fibers), and electrical stimulation, with an intravaginal or intra-anal probe. Treatment was carried out twice a week for a total of 60 min in a minimum of 10 sessions and a maximum of 40 sessions, with guidance on maintaining the perineal exercises at home. Clinical improvement was defined as at least a 50% reduction in urinary complaints at the end of therapy compared to baseline urinary complaints.	The mean age was 54 ± 12 years and 67% were women. After treatment, there was an improvement in the symptoms of urinary urgency, frequency, incontinence, nocturia, and feeling of incomplete emptying (*p* < 0.001). Overactive bladder symptom scores decreased from 10 ± 4 to 6 ± 3 (*p* < 0.001) and perineal muscle strength increased (*p* < 0.001). Urodynamic parameters improved, reducing the frequency of patients with detrusor overactivity; detrusor sphincter dyssynergia (DSD); detrusor hypocontractility, and detrusor areflexia, with positive repercussions on the quality of life of all patients.	Physical therapy for urinary incontinence effectively treated NB in HTLV-1-infected individuals, reducing urinary complaints and increasing perineal muscle strength, which reflected positively on patients’ quality of life. However, long-term follow-up and experience are required to determine the details of the used techniques.
3	Using the International Classification of Functioning, Disability, and Health as a tool for analyzing the effect of physical therapy on spasticity in patients with HAM/TSP	Rodrigues et al., 2015 [24]	This intervention study sought to evaluate spasticity in patients with tropical spastic myelopathy/paraparesis (HAM/TSP) before and after physical therapy, using the International Classification of Functioning, Disability, and Health (ICF). In total, nine individuals underwent physical therapy. Spasticity was estimated using the modified Ashworth scale. The protocol included an initial and final assessment performed after 20 sessions by the same physiotherapist. A professional was instructed to develop a program to improve gait, balance, flexibility, and muscle strength. Exercises were performed for 50 to 60 min once a week for 20 weeks. Obtained scores were converted into ICF body function scores in the b7353 category.	Active and/or passive exercises decrease spasticity, improve balance, and preserve joint integrity. The application of the physical therapy protocol improved quality of life and reduced morbidity due to the disease. Furthermore, abnormal sensory input and motor neuron activity decreased more substantially when the proprioceptive neuromuscular facilitation technique was used in this study. Therefore, strength exercises can reduce muscle spasticity and improve neural control, maintaining the observed extensibility for quadriceps muscles.	The use of ICF codes showed a greater functional independence and quality of life for patients after therapy, emphasizing the effectiveness of physical therapy in controlling spasticity and showing the value of the ICF as a tool for assessing spasticity in patients with HAM/TSP.
4	Influence of proprioceptive neuromuscular facilitation on muscle tone and range of motion in HTLV-1-infected patients with HAM/TSP	Costa et al., 2018 [9]	Investigation of five cases of physical therapy interventions to evaluate the influence of functional rehabilitation on tone and range of motion (ROM) in HTLV-1 patients with spasticity using proprioceptive neuromuscular facilitation (PNF). ROM and muscle tone were assessed using goniometry and the application of the modified Ashworth scale before and after treatment.	Patients gained ROM, especially in their lower limbs, and showed reduced hypertonia/spasticity after functional treatment.	The reduction in hypertonia increased the ROM. Thus, functional methods may be valuable for the rehabilitation of HTLV-1 patients with neurological injuries.
5	Effect of repetitive transcranial magnetic stimulation on reducing spasticity in patients with HTLV-1-associated myelopathy	Amiri et al., 2014 [25]	Pre- and post-test intervention studies aimed to evaluate the effectiveness of repetitive transcranial magnetic stimulation in reducing spasticity (as a primary outcome) and pain, in increasing muscle power, and its effect on the quality of life (as a secondary outcome) in patients with HAM/TSP. Volunteers received five consecutive days of daily sessions of high-frequency active stimulation for the motor area of their legs (20 instances of 10 pulses at 5 Hz at an intensity of 90% of the resting motor threshold for their biceps brachii muscle). Mean spasticity was defined as the mean modified Ashworth scale in lower limbs (LLLL).	In total, seven (77.8%) women and two (22.2%) men were recruited, with a mean age of 52 ± 12.67 years and a mean disease duration of 5 ± 3.94 years. A comparison of repeated measurements showed a statistically significant decrease in pain and spasticity in patients’ lower limbs. The decrease in spasticity was persistent even 30 days after the intervention but pain reduction was observed only five days after the procedure. No changes in quality of life and muscle power were detected.	Repetitive transcranial magnetic stimulation may decrease spasticity and pain in patients with HAM/TSP, and this effect can persist for a month, but it failed to influence patients’ muscle power and quality of life. This intervention can be used as adjunctive therapy in patients suffering from HAM/TSP associated with human T-lymphotropic virus type 1.
6	Proprioceptive neuromuscular facilitation in HTLV-I-associated myelopathy/tropical spastic paraparesis	Britto et al., 2014 [26]	This intervention study sought to estimate the effects of physical therapy on the functionality of patients with HAM/TSP during the stable phase of the disease using PNF and comparing two treatment application methods. In total, 14 patients with HTLV-I were randomly allocated into two groups. In group I (seven patients), PNF was applied by a therapist, facilitating the functional activities of rolling over, sitting and standing up, walking, and moving up and down stairs. In group II (7 patients), PNF was self-administered with an elastic tube, facilitating the same activities. The experiments were conducted for 1 h, twice a week, for 12 weeks. Low back pain, modified Ashworth scale, functional independence measure (FIM), and timed up and go test (TUG) were evaluated before and after the interventions.	In the intragroup assessment, low back pain significantly decreased in both groups, the functional independence measure (FIM) improved in group II, and the timed up and go test (TUG) results improved in group I.	Both PNF protocols effectively treated patients with HAM/TSP.
7	Tropical spastic paraparesis—HTLV-I-associated myelopathy: possible kinesiotherapeutic strategies to improve gait patterns in symptomatic patients	Lannes et al., 2006 [27]	This review aimed to adapt approaches for motor rehabilitation, with their respective theoretical justifications. It used a method to update the literature by works searched in databases and selected works from 1998 to 2006.	One of the most limiting aspects of the disease is lower limb weakness and spasticity with impaired gait functionality, which, in some cases, can confine patients to a wheelchair. Using a detailed analysis of the pathophysiology of the symptoms, physical therapy procedures may alleviate neurological sequelae and improve the quality of life of affected individuals.	Physical therapy, based on the proposed theoretical foundations, seems to be effective in the functional recovery of patients with HAM/TSP.
8	Physical therapy for human T-lymphotropic virus 1-associated myelopathy: literature review and future perspectives	SÁ et al., 2015 [8]	A narrative review to address the main problems associated with HTLV-1 infection that can be detected and treated with physical therapy, showing the results of clinical trials and discussing perspectives to develop knowledge in this area.	The main problems for those with HTLV-1 are pain, sensorimotor dysfunction, and urinary symptoms. It has a high impact on quality of life, and recent clinical trials involving exercise, electrotherapeutic modalities, and massage have shown promising effects.	A physical therapy approach appears to be useful for detecting specific problems related to body structures, activity, and movement-related participation in HTLV-1 infection, as well as for treating these conditions.
9	Muscle strengthening in patients with HTLV-I and its influence on functional performance: A pilot study	Figueiredo Neto et al., 2013 [28]	This intervention and analytical study sought to evaluate the impact of a muscle strengthening program in therapeutic activities on the functional performance of patients with HAM/TSP, applying a treatment protocol to strengthen muscles focused on functional activities. Participants were evaluated before and after treatment using the functional independence measure (FIM), time up and go (TUG), timed gait test (TMC), and the make test. Exercises were performed three times a week for eight consecutive weeks.	In total, 10 individuals with PET/HAM with a mean age of 45.4 years were selected, most of them women (70%), with 30% of the patients using an auxiliary device for walking. All assessment measures significantly improved, except for the make test performed for right knee flexion.	Muscle strengthening in therapeutic activities improved the functional performance of this population after eight weeks of treatment.
10	Assessment of balance in HTLV-1-associated myelopathy or tropical spastic paraparesis	Patricio et al., 2020 [29]	A cross-sectional study to analyze the most appropriate balance assessment methods for patients with HTLV-1-associated myelopathy or HAM/TSP. It related stabilometric and kinematic variables of postural oscillations with the Berg balance scale (BBS) and timed up and go (TUG) in individuals with HAM/TSP, comparing them to asymptomatic individuals. To evaluate posterior and lateral postural projection, baropodometry and the Footwork^®^ system were used, and the CVMob system was applied to the kinematic parameters.	The sample consisted of 39 subjects (predominantly women). The authors found an increase in baropodometric oscillations, total oscillation area (*p* = 0.004), anteroposterior oscillation in the left (*p* = 0.015) and right (*p* = 0.036) views, and in lateral oscillation (*p* = 0.039) in the HAM group/tsp. Moderate correlations were found between sway baropodometry and ankle angular variation, as well as with the Berg balance scale (BBS) at the three angles and the timed up and go test (TUG) for lateral sway (*p* = 0.406).	Each method has advantages and disadvantages, including cost accuracy. The best features available at no additional cost for ambulatory use are kinematic assessment using a simple smartphone camera with a free analysis software and the TUG.
11	Nintendo Wii use in patients with Ham/Tsp: randomized clinical trial	Arnaut et al., 2014 [30]	A double-blinded randomized clinical trial to verify the effect of Wii therapy as an additional therapeutic resource for patients with HAM/TSP. It was carried out with nine individuals divided into two groups—G1 performed therapeutic exercises associated with the use of Nintendo Wii games, and G2 performed only therapeutic exercises. All participants underwent pain assessment using the visual analog scale (VAS), balance assessment using the Berg scale, and answered a questionnaire on quality of life (SF-36) before and after the 10 sessions.	In the intragroup analysis, a difference was found only in the Berg Scale score on the functional capacity and emotional aspects domains of the test group (*p* < 0.05). When comparing delta scores between the groups, the emotional aspects (*p* = 0.027) and functional capacity (*p* = 0.054) domains differed between groups.	Virtual reality therapy using Nintendo Wii showed a superior positive impact on the functional exercise protocol on balance and the domains of functional capacity and emotional aspects regarding the quality of life of the participants.
12	Impact of a home exercise program on functional mobility and pain in people with PET/HAM: randomized clinical trial	Mota, 2017 [19]	A randomized clinical trial to evaluate the impact of a home exercise program with and without supervision on functional mobility and pain in people with HAM/TSP. The study was carried out on people with a defined and probable diagnosis for PET/HAM who could walk for six meters without using an auxiliary device. Participants were randomly allocated into three groups: supervised (GCS), unsupervised (GSS), and control (GC). The protocol involved muscle strengthening and stretching exercises. Assessments were performed at baseline, after 12 and 24 weeks of follow-up. The investigated variables were functional mobility, evaluated by the timed up and go test (TUG), and pain condition, assessed using the Brief Pain Inventory (BPI).	Of the 36 participants, 15 were in the supervised group (GCS), 10 were in the unsupervised group (GSS), and 11 were in the control group (GC). The sample consisted mostly of women, Black individuals, and those with low education and socioeconomic status. At baseline, they showed functional mobility restriction (TUG = 31.2 ± 18.6 s), a high prevalence of pain in their lower limbs (69.4%), and moderate lumbar (58.3%) pain. Functional performance improved in the GCS vs. GC (*p* = 0.047; post-test *p* < 0.05) and in GSS vs. GC (*p* = 0.041; post-test *p* < 0.05). The authors found no changes related to pain.	The home exercise program benefited study participants’ functional mobility in both GCS and GSS, although GCS showed the best results.
13	The effects of a home exercise program in patients with tropical spastic paraparesis/HTLV-1-associated myelopathy (PET/HAM)	Facchinetti, 2013 [31]	A case series study to evaluate the effects of a home exercise program (PED) and its adherence rate in individuals with PET/HAM. In total, 23 participants with HAM/TSP preserved gait and who had avoided exercising for at least a month participated in the study. Primary outcomes included muscle strength, maximal voluntary isometric contraction (MVIC), lower limb muscle length, pain in lumbar and lower limbs and also the use of IPEC Disability Scale, Barthel Index, and SF-36. Adherence rate and adverse events were also measured.	In the analysis, patients were divided into two groups according to the timed up and go (TUG) test (<the 20 s vs. ≥20 s). The length of the hamstring and plantiflexor muscles, lower limb MVIC, and the “Social Aspects” component of the SF-36 significantly improved in the TUG <20 s group. Individuals in the TUG ≥20 s group significantly improved the “Functional Capacity” component of the SF-36. The adherence rate was 90% overall and adverse events such as fatigue, muscle pain, and cramps were mild to moderate in intensity.	PED effectively improved some disabilities and the quality of life of individuals with PET/HAM. Such results reinforce the need for alternative strategies to the outpatient model that increase the participation of these people in rehabilitation programs.
14	Balance disorders in patients with human T-cell lymphotropic virus type 1 infection	Vasconcelos et al., 2019 [32]	A comparison of posturographic evaluation findings of asymptomatic HTLV-1-infected individuals, HTLV-1-infected individuals with HAM/TSP, and a control group database. A force platform was used to record postural sway. Analysis of variance and multivariate linear discriminant analysis were used to compare the data obtained in the three groups of participants.	HAM/TSP patients had worse balance control than HTLV-1-infected patients and the control group, but asymptomatic HTLV-1-infected patients represent a state of intermediate balance control between controls and HAM/TSP patients. TSP–posturographic parameters can be used to identify subtle changes in balance in HTLV-1 patients and monitor their functional loss.	Patients infected with HTLV-1 showed an imbalance that can be identified by posturographic parameters. Patients with HAM/TSP had balance problems, whereas HTLV-1 without HAM/TSP had a subtle impairment that was absent from clinical scales, suggesting that these patients were between healthy patients and HAM/TSP and had a risk of developing severe imbalance in postural control.
15	Postural adjustments in HTLV-1-infected patients during a self-initiated disturbance	Almeida et al., 2022 [33]	An assessment of 26 participants’ (control or infected) lower limb muscle onset and center of pressure (COP) displacements before perturbation and throughout the movement.	The semitendinosus (ST) muscle showed a delayed onset in the infected group compared to the control group. The percentage of attempts with detectable anticipatory postural adjustment was also lower in the tibialis anterior- and ST-infected groups. Moreover, the displacement of the COP in the infected group was delayed, had a smaller amplitude, and took longer to reach maximum displacement.	Patients infected with HTLV-1 have less efficient anticipatory adjustments and greater difficulty regaining postural control during the compensatory phase. Clinical assessment of this population should consider postural stability during rehabilitation programs.
16	Comparison of static balance control in HTLV-1-infected individuals with different diagnoses of TSP/HAM	Costa et al., 2022 [34]	Comparison of static balance control in HTLV-1-infected patients with different diagnoses of TSP/HAM. The sample consisted of 13 HTLV-1-infected participants and 16 healthy participants. The center of pressure was recorded using a force platform with patients’ eyes open and eyes closed. We divided the recordings into three intervals, T1 (corresponds to the first 10 s); T2 (from 10 to 45 s); and T3 (from 45 to 55 s).	In total, eight HTLV-1-infected participants were classified as probable TSP/HAM and five HTLV-1-infected participants were classified as definitely TSP/HAM. Postural instability significantly increased in patients with definitive HAM/TSP, considering the structural and global variables of body sway compared to control and probable TSP/HAM.	The severity of balance is directly related to the degree of signs and symptoms of TSP/HAM.
Legend: HAM/TSP—Tropical Spastic Myelopathy/Paraparesis ICF—International Classification of Functioning, Disability, and Health PNF—Proprioceptive Neuromuscular Facilitation LLL—Lower Limbs FIM—Functional Independence Measure TUG—Timed Up and Go Test TMC—Timed Gait Test BBS—Berg Balance Scale VAS—Visual Analog Scale GSS—Unsupervised Group GC—Control Group GCS—Group supervised BPI—Brief Pain Inventory MVIC—Maximal Voluntary Isometric Contraction PED—Home Exercise Program					

Source: Authors.

## Data Availability

Not applicable.
